# Supplementary material for: Multi-Omics Characterization of Inflammatory Bowel Disease-Induced Hyperplasia/Dysplasia in the Rag2−/−/Il10−/− Mouse Model
Source: Int J Mol Sci. 2020 Dec 31;22(1):364. doi: 10.3390/ijms22010364 (PMC7795000; doi:10.3390/ijms22010364)
Supplement: Supplementary file 1 [file ijms-22-00364-s001.zip › ijms-1054369-supplementary file.docx]

**Supplementary Information**

Multi-omics characterization of inflammatory bowel disease induced hyperplasia/dysplasia in the *Rag2^-/-^ / Il10^-/-^* mouse model

**Qiyuan Han^1^, Thomas J.Y. Kono^2^, Charles G. Knutson^3^, Nicola M. Parry^4^, Christopher L. Seiler^5^, James G. Fox^3^, Steven R. Tannenbaum^3^, and Natalia Y. Tretyakova^5*^**

^1^ Department of Biochemistry, Molecular Biology and Biophysics, University of Minnesota

^2^ Minnesota Supercomputing Institute, University of Minnesota

^3^ Department of Biological Engineering, Massachusetts Institute of Technology

^4^ Division of Comparative Medicine, Massachusetts Institute of Technology

^5^ Department of Medicinal Chemistry, University of Minnesota

***** Correspondence: trety001@umn.edu; phone: 1-612-626-3432

| **Mouse ID** | **Group** | **Inflammation** | **Edema** | **Epi  defects** | **Crypt  atrophy** | **Hyperplasia** | **Dysplasia** | **sum** |
| --- | --- | --- | --- | --- | --- | --- | --- | --- |
| 12-5624 | Control | 0.5 | 0 | 0 | 0 | 0 | 0 | 0.5 |
| 12-5625 | Control | 0 | 0 | 0 | 0 | 0 | 0 | 0 |
| 12-5626 | Control | 0 | 0 | 0 | 0 | 0 | 0 | 0 |
| 12-5638 | IBD | 1 | 0 | 0 | 0 | 0.5 | 0.5 | 2 |
| 12-5639 | IBD | 1.5 | 0 | 0.5 | 0.5 | 0.5 | 1.5 | 4.5 |
| 12-5640 | IBD | 2.5 | 0.5 | 2 | 0.5 | 1 | 2 | 8.5 |

**Table S1.** Histopathology score of proximal colon from the 6 mice used for RNA-seq and RRBS/oxRRBS analysis.

| **Gene Name** | **Protein  name** | **Log2 fold change in gene expression (Trt/ctl)** | **Methylation/Hydroxymethylation  change** | **Function** |
| --- | --- | --- | --- | --- |
| Prkch | protein kinase C eta type | 3.22 | hypomethylation | Protein Kinase |
| Tubb5 | Tubulin beta-5 chain | 1.27 | hypomethylation | Microtubules constituent |
| Zmynd15 | Zinc finger MYND domain-containing protein 15 | 1.09 | hypomethylation | transcriptional repressor |
| Mvb12b | Multivesicular body subunit 12B | -1.84 | Hypomethylation/ hypermethylation | sorting of ubiquitinated cargo protein |
| Efna3 | Ephrin-A3 | -3.47 | hypomethylation | Ligand of EPH receptor |
| Tmcc3 | Transmembrane and coiled-coil domain protein 3 | 1.13 | hypohydroxymethylation | Unknown |
| Katnb1 | Katanin p80 WD40 repeat-containing subunit B1, Katanin p80 subunit B1 | -1.25 | hypohydroxymethylation | Centrosome targeting |
| Pacsin1 | Protein kinase C and casein kinase substrate in neurons protein 1 | -1.09 | hyperhydroxymethlation | microtubule reorganization |
| Commd1 | COMM domain-containing protein 1 | -1.87 | hyperhydroxymethlation | Metabolism Regulation |
| Selenop | Selenoprotein P, SeP | -1.81 | hyperhydroxymethlation | Se transporter protein |

**Table S2.** Epigenetically deregulated genes in the IBD-CRC mouse model which are both differentially expressed and differentially methylated/hydroxymethylated. These genes are specifically highlighted as they have not been well studied in CRC previously and deserves further investigation.


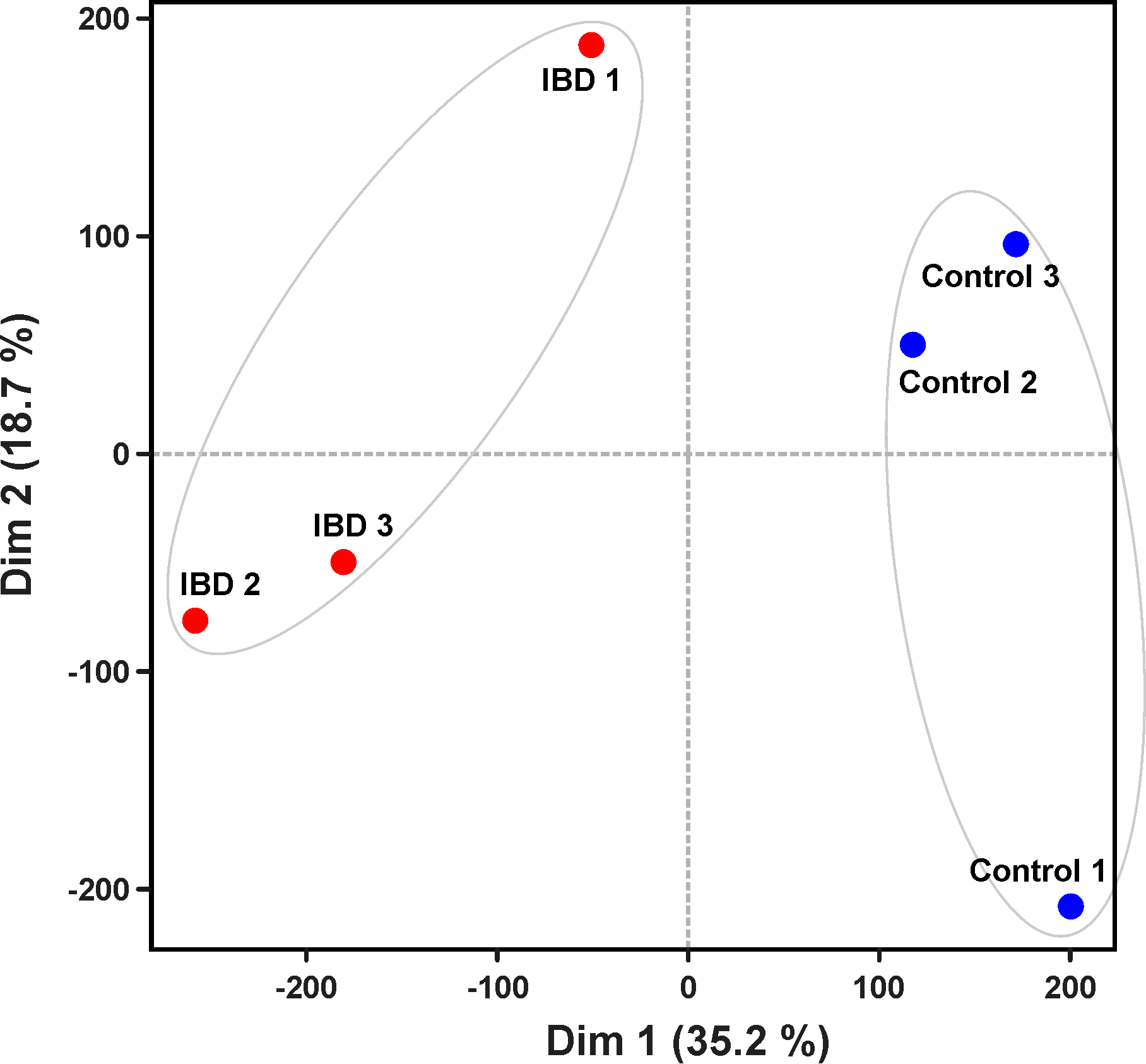


**Figure S1.** MDS plot of site specific 5mC profiling showed a clear separation between control and IBD mice.

**
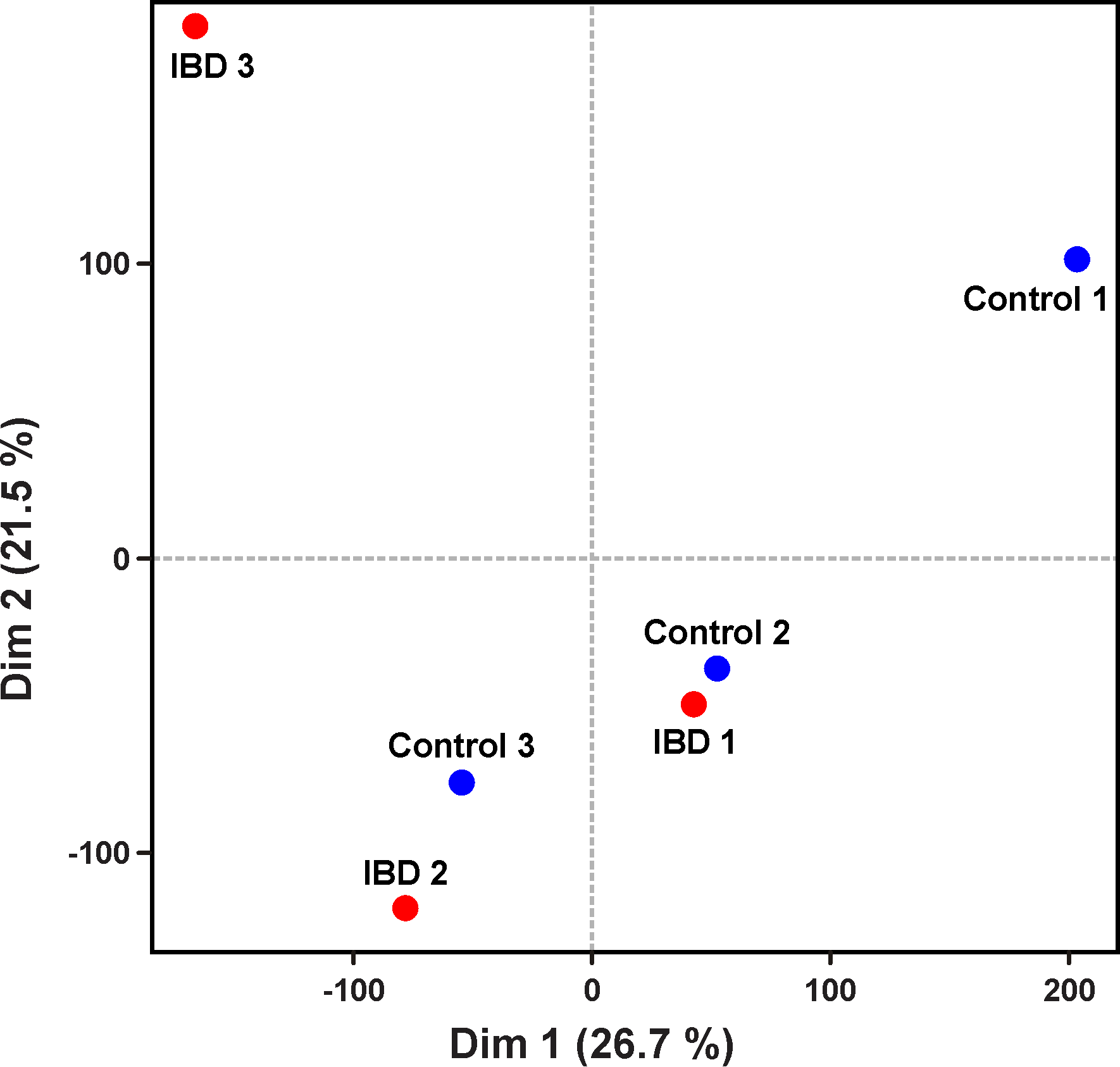
**

**Figure S2.** MDS plot of site specific 5hmC profiling in control and IBD mice.


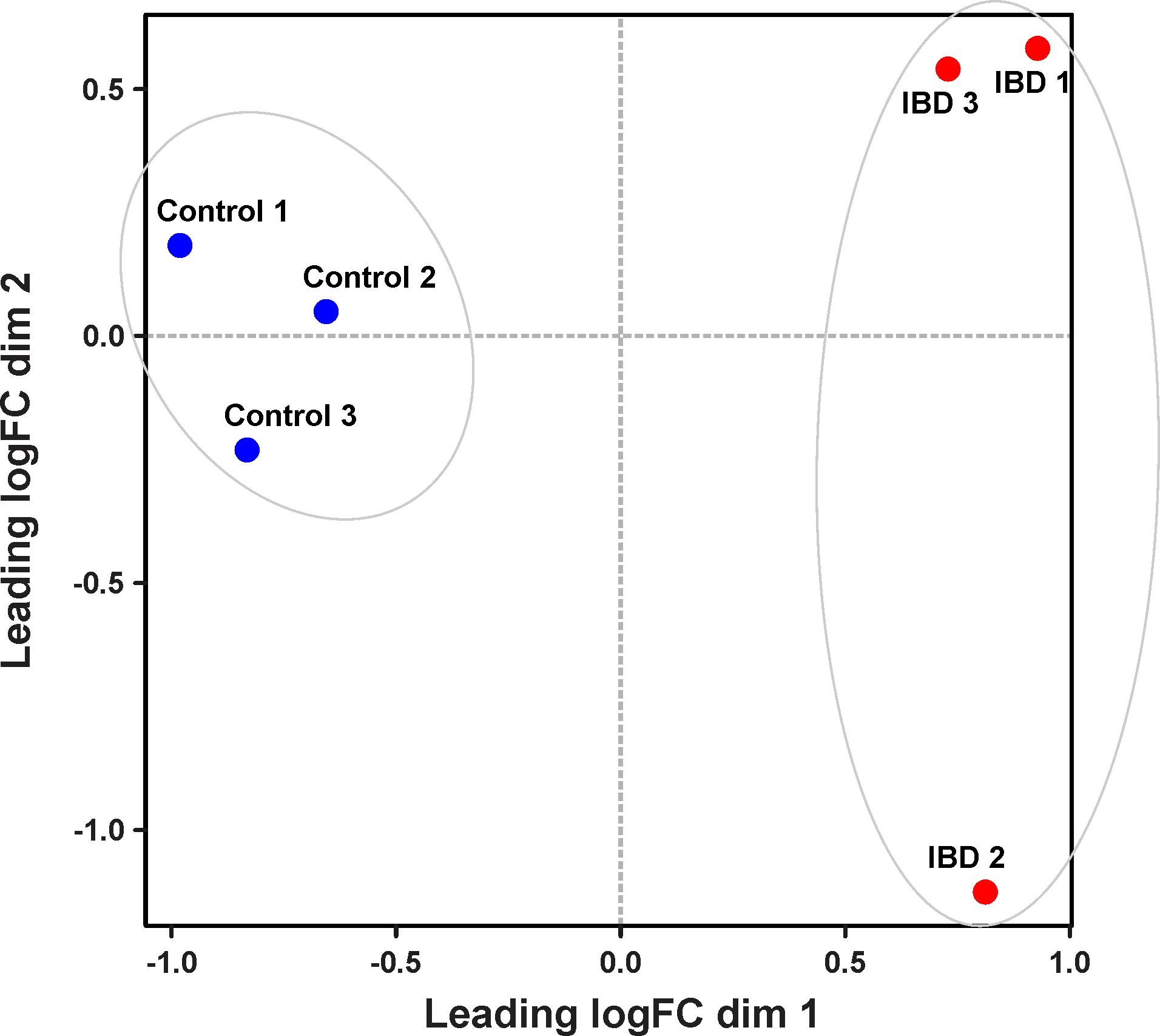


**Figure S3.** MDS plot of gene expression profiling showed a clear separation between control and IBD mice.

**Figure S4.** RT-qPCR of all *Tet* family members. The expression level was normalized to ß-Actin. N=3 in each group.
